# Dental Hobbies

**Published:** 1866-05

**Authors:** L. P. Haskell


### DENTAL HOBBIES.

BY L. P. HASKELL.

THAT *Conservative* Dentistry is the most important branch of the Art, none but charlatans would pretend to deny ; it is more desirable to retain and preserve what nature has so beautifully adapted to man's use, than to remove, and substitute therefor man's inventions. Desirable as *these* may be as the *dernier resort*, they are often resorted to from ignorance, or from selfishness.

The wonderful progress already made in conservative dentistry is certainly gratifying to every well-wisher of the profession ; and doubtless the time is coming when, to *preserve* the teeth will be the *chief*, if not the only business of the dentist. This, however, will be brought about through a thorough education of the community, in all that pertains to the health, securing, thereby, that care and attention to the teeth, together with the other organs of the body, from childhood on, that will alone secure and preserve a perfect development of them. We shall not live to see this era; but that it will come there is little doubt.

This may seem strange language for one who has confined his attention, almost exclusively, for twenty years, to the construction of artificial substitutes for these important organs ; yet such are my convictions. Still, as there is much of this work to be done, for many years to come, I offer these remarks as a preface to some words on the subject of Dental Hobbies ; and in doing this, will confine myself to such as are connected with *artificial dentistry*, leaving that numerous class, pertaining to operative dentistry, to abler hands.

Dental Hobbies are often a curse, sometimes to the profession, but oftener to the public, and are used in many instances simply to advertise and augment one's business, and *not* to benefit the patient. As, for instance, an individual says to himself, I must have some advertising dodge to bring myself before the public,—what shall it be ? I will devise some new method of inserting teeth, and in a short time the world is astonished at the anouncement of a new invention surpassing anything before known, whereby teeth can be inserted "cheaply, expeditiously, and in a manner that defies competition." He sends out, also, his agents, to dispose of office rights to other dentists, those, however, generally remote from his own immediate sphere of action, and finds plenty of just such ignoramuses as himself ready to purchase, but soon to realize that they have been *sold*, and wishing that the inventor, or his invention had been "*still-born.*" And why ? Simply because the work is *impracticable.* It may have some good points, and answer a very good purpose, provided it is never broken. But as accidents of this sort will happen, then, when the repairs become necessary, the work must be made over entirely, or so nearly torn to pieces as to be equivalent to making a new set.

No such dentures should ever be made, for it is dealing unjustly by the patient, who supposes he has a piece of work that can be readily repaired, and without costing almost as much as a new set. All such work ought to be discountenanced by the profession. I would mention, as an instance, such work as is known as the "porcelain," where no metal plate is used.

This is but one phase of dental hobbies, still another, is where the dentist takes some such method of inserting teeth as the Vulcanite, (excellent in its place,) but uses it exclusively in every class of cases. This hobby he rides to death. It should be an axiom in dentistry *that there is no one kind of work suitable for every class of cases*; and the dentist who acts upon any other principle, fails to meet the highest requirments of his calling, and does great injustice to his patient. There are cases where the Vulcanite answers a better purpose than anything else; and the man who condemns it, simply for the reason that it tends to *cheapen* dentistry, rides a very foolish, unwise hobby, and his motives do no credit to his heart at least.

Then there are those who insist that nothing but gold should be used in any case. Gold is, and ever will be, an excellent base for dentures; but he who asserts that it *alone* is suitable for such purposes, rides another hobby, in consequence of which his patients suffer, for there are numberless cases, in which either Vulcanite or Platinum are far preferable.

There, again, as to *methods* of inserting teeth: One says, always extract all the teeth remaining in the jaw, if there be but few; and others say, always leave the roots in and put your plate over them. This is a hobby which it is better that none should ride when there are so many circumstances by which one is to be governed, in such cases.

Some insist that, in *all* cases of partial sets, clasps should be used; and others insist that clasps should not be used in any case. The bests interests of the patient cannot be secured by either of these hobbies.

Then as to the *manner* of doing work ; one says, that, in order to secure the best results, an impression of the mouth must be taken, a casting made, and an impression cup swaged up, in which the impression is to be taken. Another says, take an impression in wax, and a plaster one in *that*. Another insists that wax impressions are *equal*, if not superior to any, and prides himself upon his skill in taking them. Now none of these hobbies may affect the patient particularly; for individuals may succeed well, in many cases in the use of either, but the dentist, who spends his time uselessly, and in complicated methods, and flatters himself that his is the only reliable method, and induces others to use it, without testing, in comparison with others its value, is the one, with them, to suffer from riding this hobby.

Now, as to the manner of doing work, the *simplest* method, that accomplishes the object, is the best. We are all inclined to follow, more or less, stereotyped methods, without asking the reason why, or whether another method would not be preferable. *Simplification* and not *complication* should be the aim of the dentist, always, of course, keeping in view, attainment of best results. Let me present a few illustrations of this idea.

In taking impressions, having tried all the various methods, I find that a simple plaster impression answers every purpose, and in some cases is absolutely indispensable, as for instance, partial lower sets, where the success of the denture depends upon a *perfect* impression, more than in any other case ; in some of these cases, such is the shape of the jaw, and position of the teeth, that wax cannot be used.

In making the "dies," or castings, some use a complicated flask ; whereas, if care is used in preparing the model, so that it will *drop* from the mold, (and a model will always *deliver* itself better than it can be *picked* out,) there is rarely a case that will occasion trouble ; and there again, in the worst class of cases, lower ones, "undercut" on the the lingual side, such flasks are useless. By using oiled sand, which packs better, and is less liable to break away, the difficulties are still further obviated. Sand, thus prepared, is always ready for use, and there is no danger of its "blowing."

Metals used for "dies" may be referred to in this connection. The requisites are, *hardness* without being brittle, the *least shrinkage* possible,and that will require a low temperature to melt. Tin is too soft; type metal and fusible metals too brittle; and zinc shrinks too much, and melts at too high a temperature to be used in oiled sand. Now the metal that meets all of these requirements the most fully is *Babbit* metal. It is sufficiently hard, without being too brittle, when properly prepared, has scarcely any shrinkage, and melts at a low temperature.

In swaging plates, some take great pains to swage an upper plate without cutting it open in front, spending much time to accomplish a very undesirable result. If they would consider the subject for a moment, they would see that in order to accomplish this, the plate must be cramped and contracted at the edge, leaving it in a condition in which it will be more liable to warp upon the application of heat in soldering the teeth, than if it had been cut open ; and then the plate is made absolutely stronger in its weakest point by cutting and lapping.

In the articulating of teeth, some have what might be called the *articulator* hobby, using an articulator and depending upon that, without using the patient. Now, that sets of teeth can be made to answer a purpose in that way I do not deny ; but that the best results can be attained in this way, I assert is impossible. A correct expression to a set of teeth cannot be obtained, without the use of the face itself.

It may be asked, "are not your own suggestions hobbies?" Not according to my idea of a hobby, which is a persistence in pursuing a certain method or routine, where a different one would be equally, if not more effectual, and at the same time more simple.

In conclusion, as I said before, let us aim to *simplify* processes in securing the best results, and then the young practioner will not be so often led astray, as he looks over our dental journals, for suggestions, and be misled by the various methods of doing this, that, or the other thing, some of them, perhaps long since discarded for simpler and better methods.

